# Evaluation of a Prototype Tool for Communicating Body Perception Disturbances in Complex Regional Pain Syndrome

**DOI:** 10.3389/fnhum.2013.00517

**Published:** 2013-08-28

**Authors:** Ailie J. Turton, Mark Palmer, Sharon Grieve, Timothy P. Moss, Jenny Lewis, Candida S. McCabe

**Affiliations:** ^1^Faculty of Health and Life Sciences, University of the West of England, Bristol, UK; ^2^Faculty of Environment and Technology, University of the West of England, Bristol, UK; ^3^Royal National Hospital for Rheumatic Diseases NHS Foundation Trust, Bath, UK

**Keywords:** “body perception, ” “complex regional pain syndrome, ” assessment, body schema, communication

## Abstract

Patients with Complex Regional Pain Syndrome (CRPS) experience distressing changes in body perception. However representing body perception is a challenge. A digital media tool for communicating body perception disturbances was developed. A proof of concept study evaluating the acceptability of the application for patients to communicate their body perception is reported in this methods paper. Thirteen CRPS participants admitted to a 2-week inpatient rehabilitation program used the application in a consultation with a research nurse. Audio recordings were made of the process and a structured questionnaire was administered to capture experiences of using the tool. Participants produced powerful images of disturbances in their body perception. All reported the tool acceptable for communicating their body perception. Participants described the positive impact of now seeing an image they had previously only imagined and could now convey to others. The application has provided a novel way for communicating perceptions that are otherwise difficult to convey.

## Introduction

Changes in body perception can occur following peripheral injuries, or central nervous system damage (Halligan et al., 1993; Fraser, 2002; Moseley, 2005, 2008; Lewis et al., 2007; Antoniello et al., 2010). However communicating altered body perception can be challenging for patients and assessing the changes over time is difficult for clinicians. The purpose of this project was to develop and evaluate a digital media application for communicating changes in body perception with a view to providing a useful tool for clinical practice. To achieve this aim, we required a model condition where body perception is significantly altered. Patients with Complex Regional Pain Syndrome (CRPS) provided the model for this proof of concept study. Altered body perception is commonly experienced in CRPS and has been well described (Galer and Jensen, 1999; Förderreuther et al., 2004; Moseley, 2005; Lewis et al., 2007).

Complex regional pain syndrome is a chronic pain condition of unknown etiology that usually affects a single limb. It is a syndrome that involves multiple systems with aberrant changes in vasomotor function, inflammatory mechanisms, and cortical processing (Marinus et al., 2011). These changes are probably triggered initially by a peripheral insult, but the condition quickly evolves into a centrally driven disorder for which there is currently no cure (Jänig and Baron, 2003; Marinus et al., 2011). Alongside severe pain, sufferers experience difficulty in moving the limb, disturbed proprioception, and somatosensory registration (Harden et al., 2010). Although their sensory discrimination is impaired there is often extreme hypersensitivity and painful reactions to everyday sensations such as the touch of clothing (McCabe and Blake, 2008). The limb is experienced as feeling hot or cold and other disturbances to their autonomic nervous system lead to visible changes: the limb may appear discolored with shiny skin; it may be sweaty or become more or less hairy than usual and there may be swelling. Although these changes have an impact on the appearance of the affected body part, people with CRPS often describe distressing changes in body perception which are different to the objective appearance and physical properties of their affected limb (Moseley, 2005; Lewis et al., 2007; Peltz et al., 2011). For example, a person with CRPS may describe dramatic enlargement of segments of their limb, or report perceiving sections of limb as missing. They may experience the affected limb as feeling very hot, when it is in fact cool to touch. These perceptions are usually accompanied by strong negative emotions toward the limb, which can include a desire for its amputation (Lewis et al., 2007). People with CRPS have reported they find it hard to talk about their altered body perceptions to clinicians as they do not match objective signs and they fear being disbelieved. They find it more difficult to articulate aspects of altered body perception than to describe their pain and fear being regarded as mad and having their experiences dismissed as psychosomatic (Lewis et al., 2007). Such difficulty in communicating altered body perceptions may further exacerbate their distressing emotional impact.

**Figure 1:**
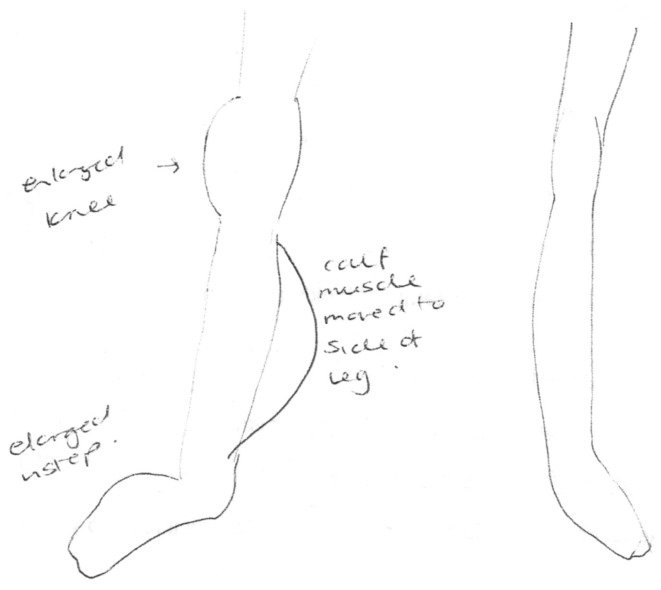
**Clinician’s drawing from description provided by participant 2 as part of the Bath CRPS Body Perception Disturbance Scale**. Notes on the drawing are: “enlarged knee,” “enlarged instep,” and “calf muscle moved to side of leg.”

Currently CRPS patients are typically asked to give verbal descriptions of their body perception in clinical interviews or if using the Bath CRPS Body perception Disturbance Scale (Lewis and McCabe, 2010). These verbal descriptions may be used by the clinician to produce a drawing or patients may be asked to draw a self-portrait (Moseley, 2005; Lewis et al., 2007; Lewis and McCabe, 2010). An example of a clinician’s drawing from a patient’s description is given in Figure 1. Although the use of drawings enable patients to describe the nature of the experiences these methods are limited by individuals’ capacity to articulate and draw well enough to adequately represent the altered body perceptions. Digital media provides a more suitable method for rendering the sensations described by people with disturbed body perception. This was demonstrated by Alexa Wright’s *After Image* project which dealt with the experience of people with amputations who experienced phantom limbs (http://www.alexawright.com/afteripg.html; Halligan, 1999). Wright manipulated photographic images of amputees to fit their described experience. In another example manipulation of digital photographs was used to represent perceived distortion of the size and shape of the face experienced by a patient with Wallenberg’s syndrome after a brainstem stroke (Rode et al., 2012). In this case quantitative data to measure the distortion was extracted from the software. However photo software isn’t quick and easy to use in the clinical setting. It is limited to one dimension and is prone to unwanted distortion of background which could influence perceptions of scale. The use of new media also offers the possibility of developing a 3D tool that would more readily enable patients to describe the nature of their altered body perception.

The aims of this project were to develop and evaluate an application that patients can use to create a 3D model of their perceived body image. This paper describes a usability and acceptability evaluation of the prototype 3D tool for communicating body perception in CRPS.

## Materials and Methods

### Specification of the body perception application

The specification for the digital media tool was determined using data from a previous exploratory study of body perception (Lewis et al., 2007), and consultation with a person with CRPS. The specification was that the tool should allow manipulation of the scaling, position, and surface texture of body segments and to display absence of parts on a model. This included the ability to lengthen and shorten limb segments, to make them thicker and thinner, and the ability to change limb position even to anatomically impossible positions. Colors and textures were to represent feelings of burning, cold, rough, smooth, and lack of substance. Finally it was also considered important to be able to view the model or “avatar” from different perspectives: front, back, left side, right side, through 360°.

The first prototype of the application satisfied all these criteria from a software perspective. It allowed modification of an avatar to depict alterations in size, shape, color, or visible surface texture of multiple body segments. Its use with consenting patients admitted to an inpatient CRPS rehabilitation program for the purposes of this research was approved by the Local NHS Research Ethics Committee.

### Recruitment of participants

Participants were recruited from a tertiary referral service for those with CRPS based in the South West of England. Inclusion criteria were: a clinical diagnosis of CRPS (Harden et al., 2010), admission to the inpatient multi-disciplinary CRPS rehabilitation program at the hospital and be able to understand and express themselves in English. Patients fitting the criteria were given the study information booklet by a member of the clinical team and were asked to contact the research nurse if they were interested. Before participation patients were required to give written informed consent to participate if willing to do so. Informed consent was obtained from all participants and is securely archived according to local NHS procedures.

### Procedure for using the application

Ten participants used the first version of the application in a single consultation with the research nurse. The nurse showed the participant the application, its capacity for altering length, thickness and position of limb segments, and the color and texture choices available for applying to the avatar’s body parts. She stressed that the illustrative meaning of colors and textures were for each individual participant to decide. Having demonstrated the scope of the software the nurse operated it in response to instructions from the participant to achieve a representation that was to their specification. For example in altering limb length she would ask the participant how long or short they wanted the limb segment to be; asking the participant to say when to stop the increase or decrease in length. Participants were asked to confirm they were satisfied with the accuracy of scaling after each manipulation.

**Figure 2:**
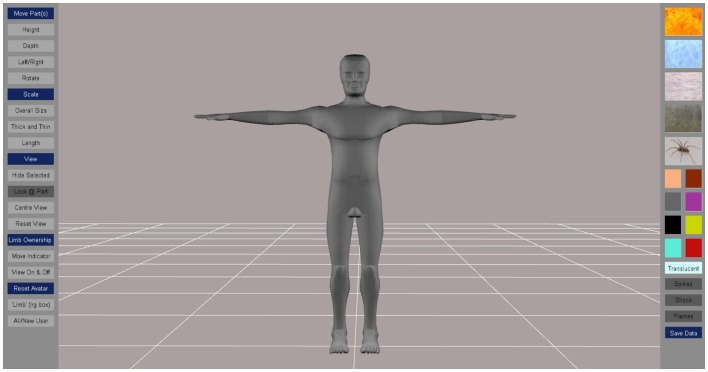
**Starting screen with options for manipulating the body perception avatar**. Menus for moving body segments (height, depth, left/right, and rotate), scaling (overall size, thickness, length), for hiding segments and changing the view are on the left hand side of the screen. Menus for colors and textures that can be applied to selected body segments are on the right hand side of the screen.

Three further participants were included after modifications to the application were made. The avatar originally only allowed the manipulation of the hand as a whole; the ability to manipulate fingers and their parts was introduced, together with the facility to represent conflicting sensations such as “flames” and ice colored “shock” representing concurrent burning and freezing cold sensations. The starting screen for this second version of the application, and its menus for manipulating the avatar, is shown in Figure 2. The procedure for the 3 additional participants was the same as for the first 10.

### Collection of evaluation data

Audio recordings were made of the participants using the application to allow interpretation of the images created. The recordings also allowed immediate reactions to the tool to be captured. Immediately after using it, participants were asked to complete a structured questionnaire which was administered face to face with the research nurse. The questionnaire had open questions to ascertain their views and experience of using the tool. The questionnaire was modified after the first 7 participants had completed the evaluation to include a rating out of 10 to determine how good a representation participants’ thought the created image was and to explicitly ask whether using the tool caused increased pain and distress (see Appendix). This version of the questionnaire was used with six participants.

### Data analysis

Images were saved anonymously to protect the identity of participants. Questionnaire responses were all given an anonymized study identity code. The questionnaire responses were collated and the audio interviews were transcribed. They were analyzed for their content to determine acceptability and usability of the tool under the following headings: (i) the ability of the body perception tool to represent participant’s experience, (ii) their reactions to using the tool, (iii) limitations and aspects for refinement.

## Results

### Participants

Reflecting the CRPS population, participants were predominantly female (10 female). Ages ranged from 24 to 64 years (median 54), and CRPS duration ranged from 6 months to 7 years (median 14 months). Ten had an upper limb affected and three a lower limb (one participant had both upper and lower leg on one side affected). The characteristics of each participant are listed in Table 1.

**Table 1:** **Characteristics of participants**.

Participant	Gender	Age	Duration CRPS (months)	Limb(s) affected
1	F	24	6	Right upper limb
2	F	57	72	Right lower limb
3	F	58	10	Left hand
4	F	56	14	Left hand
5	F	64	14	Right hand
6	F	55	10	Left upper limb
7	F	28	29	Right lower limb
8	F	54	61	Right upper & lower limb
9	F	60	12	Right wrist
10	M	27	21	Left lower limb
11	M	26	43	Right arm
12	F	44	13	Left arm
13	M	53	48	Right hand

### The ability of the body perception tool to represent participant’s experience

**Figure 3:**
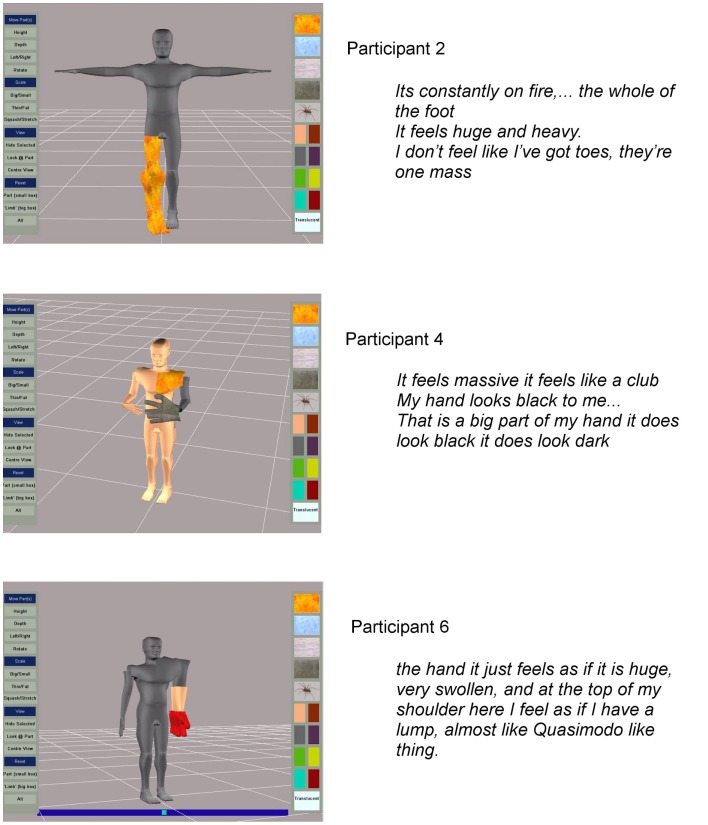
**Examples of images captured with participant’s verbal descriptions**.

The participants produced powerful images of the disturbances in body perception they experienced. Some examples supported with quotes of participants’ verbal descriptions are given in Figure 3. Participant identification codes on the figures, and given in brackets after quotes used in the text, relate to the participant numbers in Table 1.

Alterations in scaling were common with participants feeling that limb parts were either larger or smaller than normal and these perceptions were illustrated on the computer model (examples are given in Figure 3). Participants liked the application’s ability to scale and distort body parts. However, 5 of the 10 participants tested with the first prototype wanted more detailed representation of the hands.

Pain and altered somatosensation were illustrated by the application of a single color or texture in the prototype. Participants reported liking the colors, particularly the fire effect which they used to represent burning pain. Nevertheless it was not uncommon for participants to report dynamic sensations, pins, and needles or contradictory sensations, which could not be portrayed in the first version of the software. Participants frequently reported an extreme burning sensation but this was sometimes experienced with a contradictory cold sensation in the affected region.

*The electric shocks that go up and down it are obviously not there, so I don’t know how you could represent those sort of things*. [Participant 6]

*My hand feels as if it’s absolutely on fire and then if somebody touches it, it feels cold, and this pins and needles. I don’t know how to represent that. It’s like numb but I can feel it*. [Participant 5]

Regarding the avatar, participants liked the facility to view and manipulate it from different perspectives. Some participants, though not all, liked the fact the manikin was not portrayed as a human; preferring the impersonality of the schematic figure.

Interviewer: … at the moment it’s kind of a grey figure I mean are you happy with that, do you think it would be an improvement to make it look more human

*No, because it’s not, it if were more human it’s going to be a more direct personal thing*. [Participant 3]

*I think it’s kind of better you don’t feel as pushed as if maybe you saw like a human being*. [Participant 1]

*It probably would be better if it was more of a human form because at the moment it’s very robotic looking*. [Participant 6]

### Participants’ reactions to using the tool

In response to the question “Did you find using the body perception application an acceptable way to communicate how you view or feel about your limb or body parts?” All participants reported using the tool was a good method for communicating their body perception; both for themselves and for helping the clinician to understand patients’ body perceptions. The last seven participants were asked to rate out of ten how satisfied they were with the images they created: three gave a rating of 7, one 8, one 9, and one 10. All participants were unanimous in the view that using the application was better than the standard interview about body perception experienced earlier in their admission. They appreciated the application was much more adaptable than a clinician’s sketch. They found the application easy to use in consultation with the research nurse. One participant commented that they felt the process of the consultation with the nurse led to a more honest representation than might have resulted through independent use of the application.

*I don’t think for myself it would work if I were expected to use it, but I think getting somebody else to do it, you can explain more. I think that if I did it I’d perhaps not be quite as honest as telling you about it. I felt more honest being open with you and telling you exactly what, I sat there and I did it I might have made that little bit smaller but that’s exactly how I see it*. [Participant 8]

Some participants expressed the idea that the image made them realize the extent of their altered body perception. Some participants expressed surprise at how they were able to depict their affected region using the system and at their own reaction to seeing the representations they created.

*It was like quite bizarre seeing a picture of how exactly I felt as a person, cause I’ve never had that opportunity of looking at that like that … for me as I say to visualize that’s how I feel. I felt a bit emotional, but the more I’m looking at it, it’s only because I’m sitting here thinking that is exactly how in my mind’s eye what I look like, so it was a bit of a shock I suppose*. [Participant 8]

*This is much more true to life, I’m not saying I’m going to have a panic attack but this is making me think a lot more about my hand than any talking about it*. [Participant 4]

*It makes you see how distorted your vision of your own body is, of your limb especially*. [Participant 6]

*In your head I haven’t said this word but I’ve felt this, you feel freakish, so you look at that and you think yeah that is how I feel*. [Participant 2]

Pain may be increased by dwelling on the affected body parts (Lewis and McCabe, 2010), so the last six participants were asked if using the tool increased their pain or distressed them. Two instances of increased pain were reported.

Interviewer: *Did using the tool change your pain in any way?*

Participant: *To be honest I know this is going to sound crazy probably but my hand is absolutely killing me; I don’t know why*. [Participant 8]

The same participant reported that though she didn’t like it, she was not distressed and there were benefits.

*No, I don’t think I’ve got a bad feeling from doing this, it’s not a bad feeling it’s just to me looking at that puts it into perspective what I’ve got. It’s just I don’t know how to explain it. It looks in human form exactly how I feel and I’ve never had that. I’ve sat and said this hand feels longer and feels wider from there. I know how I can see it but this is the first time somebody else has*. [Participant 8]

### Limitations and aspects for refinement

**Figure 4:**
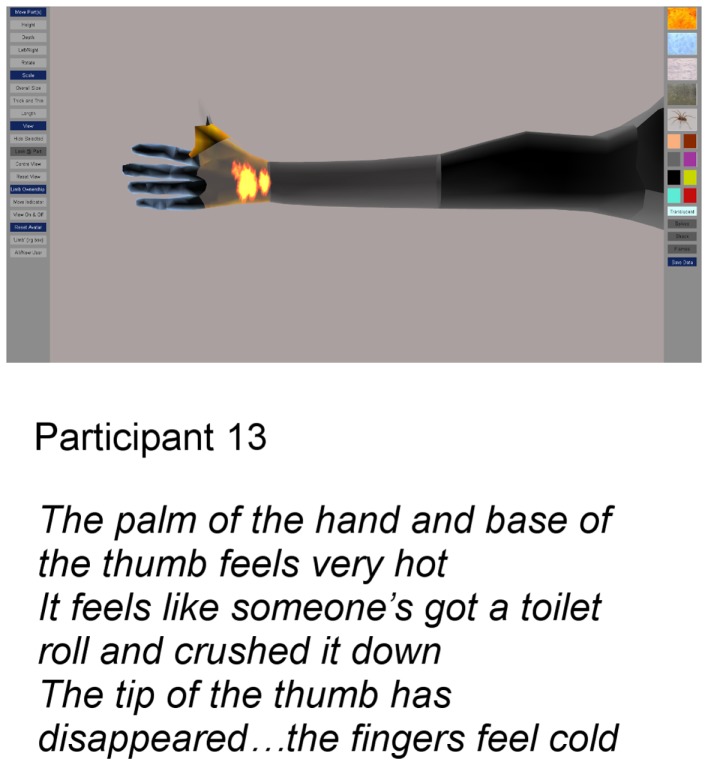
**Image produced after additional features were added to the application: particle texture effects and fractionation of digits**.

Main limitations of the prototype identified by the first 10 participants were the lack of detailed representation of the hands and the lack of ability to represent more than one sensation in a single body or limb segment. Program modifications allowing finer manipulation of individual fingers and additional surface options to portray conflicting temperatures and shooting sensations were made and version 2 was tested on the three last participants. All three used the refinements to fractionate the hand and the additional textures and found them to be acceptable features (for an example see Figure 4).

Other suggestions, not yet implemented, were to add in a representation of the sensation of compression of the limb segment and animation of perceived movement or tremor.

## Discussion

This is the first time CRPS evoked disturbances in body perception have been captured in such a graphical manner. The quality of the graphics enhanced the reality of the image thereby helping participants to fully convey to themselves and others how altered their bodies seem to them. Participants described the positive impact for them of now seeing an image of a limb that they had previously only imagined and could now convey to others. The experience of viewing the image resulting from their visualization elicited some interesting reactions. This was apparent in the surprise that was often expressed and in the pain experienced by some participants. These reactions to visualizing body perception are not confined to use of the body perception tool. It has been reported before when mental visualization was used to help patients to verbally describe their body perception (Lewis et al., 2007). Constructing an acceptable representation on the screen provided a more powerful and adaptable means to communicate body perception than the use of drawings and may also provide a method to help patients to accept the conflicting perceptions of the body they experience.

A limitation of the study was that consultations with the research nurse and the administration of the questionnaire were not completely independent of the application’s developer. The software developer was present (with the consent of the participant and approval of the Research Ethics Committee) in many cases. This was because he wanted responses first hand and initially it was to help train the nurse in using the tool. This lack of independence in the evaluation could have led participants to be more positive about the application than they might otherwise have been. However both the nurse and the software developer stressed their desire for the participant’s honest opinions in order to establish the acceptability of the body perception application to patients and for identifying aspects that needed to be changed.

Areas for improvement to the application were identified, for example animation of perceived involuntary movements and creating more sophisticated depictions of sensation. However future use of the application needs to be considered before adding greater levels of detail and complexity. With increasing use of telemedicine, future versions of the application could be made to enable the software to be used by patients independently of clinicians in their own homes over the internet. The participants tested seemed to like using the application with the nurse; perhaps because it was new to them and may have appeared complicated. One of the participants even expressed the view that she was likely to have been less honest if she had used it on her own. However with the increasing use of finger activated tablets development of the tool using touch screen operation that is intuitive and user-friendly is important for its future development. Manipulation using finger drags and taps to turn the avatar, enlarge and shrink parts, and drop on textures and color will need to be robust for use on these smaller screens. The operations will have to be easy to complete with the non-dominant hand, since in some individuals their condition will have affected dexterity in their preferred hand. Further careful evaluation of independent usability and comparison of results between methods of delivery will be needed to determine reliability.

There is also further work to do in exploring the best form of the avatar for patients to represent their disturbed body perception. Previous research using body image morphing techniques with fit healthy people and with obese people has indicated that body perception is influenced by the form of the image presented (Stewart et al., 2003; Johnstone et al., 2008). Our participants with CRPS expressed mixed views of the anonymous gray avatar with some participants preferring its impersonal nature. Preferences for the human-likeness or individual’s likeness to an avatar may be influenced by age, culture, and emotional resilience (Walters et al., 2008). People with CRPS may experience increased pain or distress when using the tool if the avatar is created to look very like them.

Further development and evaluation is in progress to determine the use of the body perception application with people with stroke. A significant prevalence of phantom limb in the form of postural illusions of limb position has been found in people with stroke (Antoniello et al., 2010). Assessment of body perception is not routine in clinical practice but strong similarities in clinical presentation (i.e., motor and sensory and spatial cognition impairments), and in the findings from studies of cortical changes between CRPS and stroke suggest that some common disturbances in body perception may be found (Acerra et al., 2007).

Since there is potential to “repair” distressing body perception using rehabilitation interventions (Flor et al., 2001; Flor, 2002), it is conceivable that digital images of body perception could be used, not just for communicating body perception, but also as part of a treatment. Future versions of the application might allow virtual movements and sensory experiences and with the introduction of interfaces such as Microsoft’s Kinect, it would be possible to represent movements of individuals in ways that might affect their body perception or reduce their pain (Huang et al., 2006; Slater et al., 2009).

Another potential function of the body perception application is to quantify changes in predominant features in response to treatment. Other investigators have taken measurements of scaling from photo software. Vector deviations of image manipulations of a patient’s representation, relative to a reference photograph, were obtained to measure perceived size changes of one side of his face (Rode et al., 2012). The consistency of an individual’s representation of size on an image or avatar manipulated on a screen, as well as sensitivity to change in the patient’s body perception need to be tested. The reliability of scaling measurements extracted from the manipulated avatars is currently being investigated in a pilot study before testing in a larger sample. Pain and body perception are positively correlated (Lewis and Schweinhardt, 2012) and reliable measures of change in body perception might provide insight into mechanisms of interventions and the natural history of CRPS.

## Conclusion

This proof of principle study has shown that the body perception tool provides a powerful vehicle for communicating and representing changes in body perception.

We envisage that this tool could extend beyond being a very useful communication device between patients and clinicians and also become a meaningful process measure and an interactive tool for intervention.

## Conflict of Interest Statement

The authors declare that the research was conducted in the absence of any commercial or financial relationships that could be construed as a potential conflict of interest.

## Appendix 1

### Questionnaire

**Table:**

1	How did you find your experience of using the tool – did you find it an acceptable way to communicate how you view or feel about your limb or body parts?
2	Do you think the tool enabled you to describe your body perception better than you would in the usual interview with the occupational therapist?
3	What features of the computer program did you like?
4	Is there anything you didn’t like about using the tool?
5	How could the tool be improved?

### Patients 8–13 were given a revised version of the questionnaire which added these questions

**Table:**

6	a) Did using the body perception tool change your pain in any way?
	b) Was this experience any different, in terms of pain, to the one you had during your body perception interview with the occupational therapist?
7	a) Did using the body perception tool cause you any distress?
	b) Was this any different, in terms of levels of distress, to your experience during your body perception interview with the occupational therapist?
8	Was the resulting image a good representation of how your limb (or body parts) look and feel to you? On the scale below put a mark to represent how happy you are with the image you made (0 = Not happy at all with image and 10 = image is exactly as I would like)

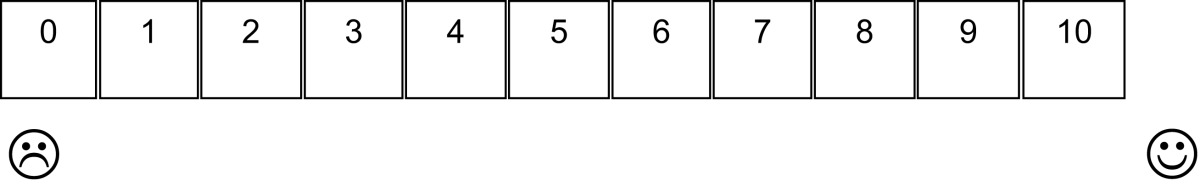
	
9	Was the body perception application easy to use with the help of the research nurse?
10	Do you have any other comments about the application or your experience of using it?
